# Epitranscriptomic(N6-methyladenosine) Modification of Viral RNA and Virus-Host Interactions

**DOI:** 10.3389/fcimb.2020.584283

**Published:** 2020-11-24

**Authors:** Hasan Imam, Geon-Woo Kim, Aleem Siddiqui

**Affiliations:** Division of Infectious Diseases, Department of Medicine, University of California, San Diego, La Jolla, CA, United States

**Keywords:** viral epitranscriptomics, m^6^A-writer, m^6^A-eraser, m^6^A-binding protein, m^6^A modification

## Abstract

N6-methyladenosine (m^6^A) is the most prevalent and internal modification of eukaryotic mRNA. Multiple m^6^A methylation sites have been identified in the viral RNA genome and transcripts of DNA viruses in recent years. m^6^A modification is involved in all the phases of RNA metabolism, including RNA stability, splicing, nuclear exporting, RNA folding, translational modulation, and RNA degradation. Three protein groups, methyltransferases (m^6^A-writers), demethylases (m^6^A-erasers), and m^6^A-binding proteins (m^6^A-readers) regulate this dynamic reversible process. Here, we have reviewed the role of m^6^A modification dictating viral replication, morphogenesis, life cycle, and its contribution to disease progression. A better understanding of the m^6^A methylation process during viral pathogenesis is required to reveal novel approaches to combat the virus-associated diseases.

## Introduction

Methylation at N6 position of adenosine (m^6^A) is the most well-characterized and one of the most abundant internal modifications of cellular mRNAs, viral transcripts, long noncoding RNAs (lncRNAs), and microRNAs (miRNAs) (Fu et al., 2014; Meyer and Jaffrey, 2014). The term epitranscriptomic was coined to indicate this dynamic, reversible, co-transcriptional process of RNA synthesis. Pioneering studies in 1970s established m^6^A as the major form of internal methylation on mammalian mRNA, which occurs in the sequence-context of (G/A)(m^6^A)C (Desrosiers et al., 1974; Perry and Kelley, 1974; Schibler et al., 1977; Wei and Moss, 1977). With the recent advent of highly sensitive detection methods of antibody-mediated immunoprecipitation combined with high throughput sequencing methods leading to the identification of m^6^A sites in the transcriptome, functional significance of m^6^A has been intensely investigated (Dominissini et al., 2012; Meyer et al., 2012). These studies significantly intensified for viral genomic RNAs and transcripts of DNA viruses form the focus of this review.

The key principles revealed by the m^6^A-mapping studies have shown that m^6^A is a selective modification, based on its enrichment in certain mRNAs. There is a single m^6^A site in most of the m^6^A-modified mRNAs, where some mRNAs may contain 20 or more m^6^A sites. Transcriptome-wide m^6^A site mapping has provided greater details on its localization and prominence, revealing its frequency in thousands of transcripts. In different tissues and cell lines, most of the m^6^A sites appear to be constitutive and are distributed among the corresponding mRNAs in a very similar way (Dominissini et al., 2012; Meyer et al., 2012; Schwartz et al., 2014; Linder et al., 2015). In human and mice transcriptomes, m^6^A sites are distributed in a unique way preferentially around the stop codons and enriched at 3’ untranslated regions (3’ UTRs) (Dominissini et al., 2012; Meyer et al., 2012). The structure of a specific gene and abundance of m^6^A are correlated because the presence of long internal exon can be a strong inducer for m^6^A addition in the transcribed mRNA (Dominissini et al., 2012; Batista et al., 2014; Geula et al., 2015; Ke et al., 2015; Ke et al., 2017). Also, specific gene categories that regulate the development and cell fate specification may be associated with mRNAs containing an unreasonably high level of m^6^A (Dominissini et al., 2012; Meyer et al., 2012; Geula et al., 2015). After various cellular stresses or in different developmental states, changes in m^6^A levels have been detected in the 5’ UTRs of a variety of mRNAs (Dominissini et al., 2012; Meyer et al., 2012; Yoon et al., 2017). m^6^A modification can control the outcome of methylated RNAs at multiple steps, including cap-dependent translation, RNA splicing, mRNA stability, and miRNA biogenesis and m^6^A modified RNAs participate in many biological processes, like cellular reprogramming, stem cell differentiation, fertility, stress response, circadian cycle and cancer (Dominissini et al., 2012; Fustin et al., 2013; Li and Mason, 2014; Alarcon et al., 2015b; Chen et al., 2015b; Meyer et al., 2015; Zhou et al., 2015; Zhao and He, 2017). m^6^A modification not only exists in the RNAs of eukaryotic cells but also in genomic RNAs of viruses and of transcripts of DNA viruses. Deregulation of m^6^A modification is related to the diseases caused by pathogenic viruses. N6 methylation was discovered by Robert Perry in 1974 (Perry and Kelley, 1974). Early studies identified m^6^A residues in the mRNAs of Simian virus 40 (SV40), Influenza A virus (IAV), Adenovirus, Avian sarcoma virus, and Rous sarcoma virus (Hashimoto and Green, 1976; Krug et al., 1976; Beemon and Keith, 1977; Canaani et al., 1979). Biochemical analysis of different Influenza virus mRNAs showed a varied distribution of m^6^A in different hemagglutinin (HA) mRNAs (Narayan et al., 1987). But it was not possible to explore the function of m^6^A in RNA splicing and translation due to insufficient understanding of the experimental conditions and relevant knowledge of m^6^A for decades. The transcriptome-wide m^6^A mapping coupled with the development of the N6-methyladenosine-sequencing (m^6^A-seq) method, revealed this widespread RNA modification linked to various biological functions including differentiation, sex determination, metabolism, stress response, virus infections and cancer (Dominissini et al., 2012; Meyer et al., 2012; Gokhale et al., 2016; Kennedy et al., 2017). m^6^A-seq was followed by other newly developed techniques like PA- m^6^A-seq, miCLIP, m^6^A-LAIC-seq, microarray, and SELECT (Chen et al., 2015a; Li et al., 2015; Linder et al., 2015; Molinie et al., 2016; Xiao et al., 2018). Development of these tools led investigators to elucidate the role of m^6^A in viral epitranscriptomics. Other forms of epitranscriptomic modifications including, 5-methylcytidine and N4-acetylcytidine were also reported in viral genome but their functional significance remains elusive (Courtney et al., 2019; Tsai et al., 2020).

m^6^A modification in different pathogenic viruses is increasingly being studied in recent years to reveal its role in the regulation of viral life cycles. m^6^A modification can influence specific gene expression involved in viral life and can play a role in the inhibition or promotion of different pathogenic virus replication. In this review, we will summarize the emerging roles of m^6^A modifications described in the recent literature with a focus on viruses and discuss their functions and associated mechanisms related to the biological processes of different virus infections.

## Cellular Machinery Associated With m^6^A Modification

### Writers for m^6^A Methylation

**Figure 1:**
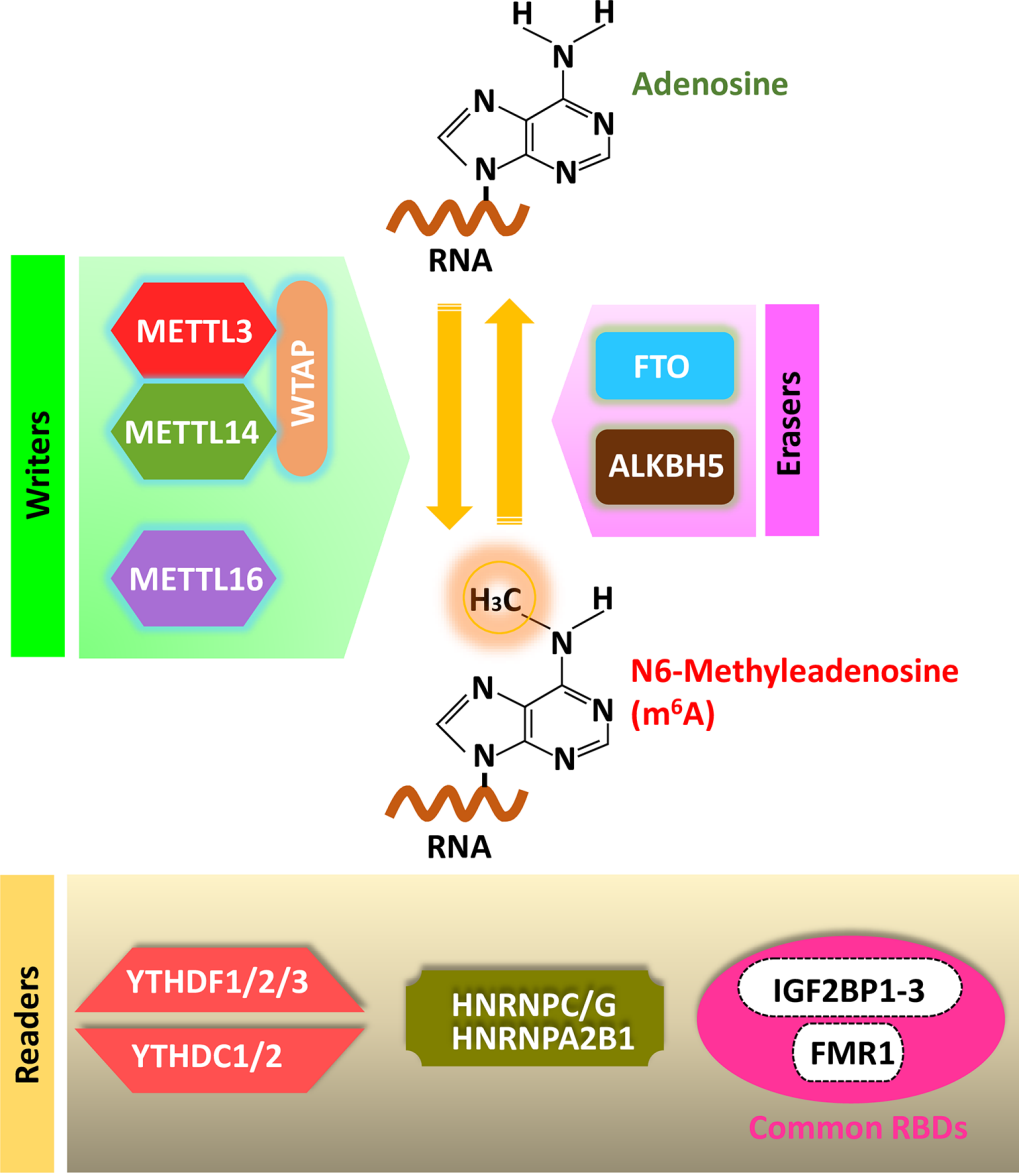
Cellular m^6^A machinery: Writers, Erasers and Readers. Writer complex is composed of core subunits METTL3 and METTL14 with some additional adaptor proteins. METTL16 is also known as writer. Erasers: FTO and ALKBH5 are the known m^6^A erasers. Readers: YTH-domain containing proteins (YTHDF1-3, YTHDC1-2) directly recognize m^6^A- containing RNAs. A local structure disrupted by the presence of m^6^A could favor RNA-binding events of several heterogeneous nuclear ribonucleoproteins including HNRNPC/G and HNRNPA2B1. RNA binding proteins including IGF2BP1-3 and FMR1 prefer m^6^A-modified RNAs.

Mammalian genome encodes various methyltransferases to establish m^6^A modification in distinct RNAs (**Figure 1**). Methyltransferase-like 3 (METTL3) and methyltransferase-like 14 (METTL14) form a stable core complex that co-transcriptionally installs m^6^A on mRNA (Bokar et al., 1997; Liu et al., 2014). In METTL3-METTL14 heterodimer, METTL3 is the enzymatic component and METTL14 acts as an allosteric activator to facilitate RNA binding (Sledz and Jinek, 2016; Wang P. et al., 2016; Wang X. et al., 2016). Wilms tumor 1-associated protein (WTAP), Vir like m^6^A methyltransferase associated (VIRMA), Zinc finger CCCH-type containing 13 (ZC3H13), and RNA binding motif protein 15/15B (RBM15/15B) are the additional subunits that contribute to the activity and specificity of m^6^A writers. WTAP binds to METTL3-METTL14 heterodimer and is necessary for the optimal substrate recruitment and localization of METTL3/14 complex. VIRMA is critical for the deposition of m^6^A specifically to the 3’ UTR, ZC3H13 facilitates nuclear localization of the writer complex and RBM15/15B binds to U-riched regions and can expedite methylation of certain mRNAs (Zhong et al., 2008; Ping et al., 2014; Patil et al., 2016; Wen et al., 2018; Yue et al., 2018). METTL3 forms a stable dimer with METTL14 while interacting with WTAP and was localized in the nuclear speckles of HeLa cells, although its cellular distribution can differ between cell lines and its redistribution can be induced by cellular stress. In both METTL3 and WTAP, functional nuclear localization signals (NLS) have been identified and mutations created in the key residues abolished preferential nuclear localization of ectopic METTL3 and WTAP. In multiple human cancer cell lines, a fraction of METTL3 protein has been observed in the cytoplasm at various proportions and the possible reasons could be due to varied amounts of protein abundance ratios between METTL3 and METTL14 with other adaptor subunits. Post-translational modifications (PTMs) could be the other reason which can change the interaction between METTL3 with its partner proteins, leading to cytoplasmic existence (Bokar et al., 1997; Ping et al., 2014; Alarcon et al., 2015b; Chen et al., 2015b; Lin et al., 2016; Barbieri et al., 2017; Knuckles et al., 2017; Xiang et al., 2017; Choe et al., 2018; Scholler et al., 2018).

m^6^A methylation is transcript-specific and the writer complex is recruited at desired chromatin loci through transcription factors and/or epigenetic marks (Shi et al., 2019). In response to stimuli and stress, a subset of m^6^A sites appears dynamic (Dominissini et al., 2012; Meyer et al., 2012). METTL3 was localized in the heat-shock genes in chromatin after heat shock and when the stress was finished m^6^A formation cleared from those heat-shock RNAs. METTL3/14 heterodimeric complex was localized in UV-induced damaged sites upon DNA UV damage, co-occurring with enhanced m^6^A intensity. A specialized adaptor protein might target the m^6^A writer in a distinct set of genes in the chromatin, which can lead to transcript-specificity of m^6^A methylation (Barbieri et al., 2017; Knuckles et al., 2017; Xiang et al., 2017).

Another m^6^A writer, methyltransferase-like 16 (METTL16), which forms a homodimer, installs m^6^A in a different sequence and structure context than METTL3/14 complex. METTL16 has a preference for UAC(m^6^A)GAGAA sequence in the bulge region of a stem-loop structured RNA and it has been confirmed by *in vitro* methylation selection assays, truncation/mutation tests, and a crystal structure of METTL16 protein with its RNA substrates. METTL16 has two authenticated substrates: U6 small nuclear RNA (snRNA) and a hairpin (hp1) in the 3’ UTR of human methionine adenosyltransferase 2A mRNA (*MAT2A*) which encodes for S-adenosyl methionine (SAM) synthetase (Liu et al., 2014; Pendleton et al., 2017; Doxtader et al., 2018; Mendel et al., 2018). ZCCHC4 is the recently identified m^6^A writer, mediates the methylation of A4220 on 28S rRNA within an AAC motif, and interacts with a subset of mRNAs (Ma et al., 2019).

### Erasers for m^6^A Methylation

m^6^A demethylases fat mass and obesity-associated protein (FTO) and AlkB homolog 5 (AlkBH5) can reverse m^6^A methylation *via* active demethylation are considered as erasers (**Figure 1**). The first identified RNA demethylase, FTO can remove the methyl group of m^6^A in mRNA and *N* ^6^,2’-O-dimethyladenosine (m^6^Am) in a portion of mRNA, both *in vitro* and inside the cells (Adams and Cory, 1975; Wei et al., 1975; Jia et al., 2011; Fu, 2012; Fu et al., 2013; Mauer et al., 2017). m^6^Am is a terminal modification at mRNA cap that exists in higher eukaryotes (Sun et al., 2019). Initially, FTO was described as a nuclear protein which contains a nuclear localization signal (NLS) in the N terminus and partially co-localize with nuclear speckles. But FTO was shown to localize both in the nucleus and cytoplasm in certain cell lines and its cellular distribution differs among other mammalian cell lines. A large portion of FTO protein localizes in the cytoplasmic part of certain acute myeloid leukemia (AML) cell lines, where it can demethylate cap-m^6^Am and up to 40% of all mRNA m^6^A. Also, 5% to 10% of mRNA m^6^A are demethylated by FTO in HeLa and HEK cell lines (Sanchez-Pulido and Andrade-Navarro, 2007; Jia et al., 2011; Gulati et al., 2014; Aas et al., 2017; Li et al., 2017; Su et al., 2018; Wei et al., 2018).

AlkBH5 is the second known RNA demethylase of m^6^A modification. AlkBH5 can regulate the nuclear export of target RNAs and this function is affected by the demethylation activity. In certain transcripts demethylation of 3’ UTR is mediated by AlkBH5. AlkBH5 has been reported to facilitate hypoxia-induced HIF-dependent breast cancer stem cell phenotype, regulation of glioblastoma proliferation, and tumorigenesis through the AlkBH5-FOXM1 pathway, and modulation of splicing and stability of long 3’ UTR mRNAs in male germ cells. AlkBH5 and FTO expression patterns are different and they participate in different biological pathways and interact with different protein partners (Zheng et al., 2013; Zhang et al., 2016; Zhang et al., 2017; Tang et al., 2018).

### Readers for m^6^A Methylation

YT521-B homology (YTH) domain-containing proteins (YTHDF1-3 and YTHDC1–2) can bind m^6^A containing motifs and are known as m^6^A readers (**Figure 1**). YTHDF2 and YTHDF3 proteins were first recovered from an *in vitro* m^6^A-RNA pull-down experiment (Dominissini et al., 2012). Cytoplasmic YTHDF2 protein can recruit the CCR4-NOT (C-C motif chemokine receptor 4 - negative on TATA-less) deadenylase complex and promotes the degradation of target transcripts (Wang et al., 2014; Du et al., 2016). But, the other two cytoplasmic m^6^A readers YTHDF1 and YTHDF3 can recruit translation initiation factors and support the translation of target transcripts (Wang et al., 2015; Shi et al., 2017). YTHDC1 protein, an m^6^A reader localized in the nucleus performs several roles by enabling the nuclear export of mRNA, accelerating the decay of certain transcripts, and regulating mRNA splicing by recruiting certain splicing factors (Xiao et al., 2016; Roundtree et al., 2017b; Shima et al., 2017). Another nuclear m^6^A reader protein YTHDC2 regulates spermatogenesis and mediates both mRNA stability and translation (Hsu et al., 2017).

Another group of m^6^A readers can preferentially bind m^6^A-containing RNAs by utilizing common RNA binding domains (RBDs) such as K homology (KH) domains, RNA recognition motif (RRM) domains, and arginine/glycine-rich (RRG) domains. RNA protein interaction can be modulated by the presence of m^6^A, named “m^6^A-switch”, which can remodel the local RNA structure (Liu et al., 2015). Several heterogeneous nuclear ribonucleoproteins (HNRNPs) can regulate alternative splicing or processing of target transcripts, which includes HNRNPC, HNRNPG, and HNRNPA2B1 (Alarcon et al., 2015a; Liu et al., 2015; Liu N. et al., 2017; Wu et al., 2018). Fragile X mental retardation 1 (FMR1) protein binds m^6^A-containing RNA and contains three KH domains and one RGG domain. FMR1 interacts with m^6^A readers YTHDF1 and YTHDF2 and can modulate both RNA translation and stability (Edupuganti et al., 2017; Zhang et al., 2018). Although, presence of YTHDF2 protein makes the m^6^A containing mRNA unstable but another class of m^6^A reader, insulin-like growth factor 2 mRNA-binding proteins (IGF2BP1–3), stabilized the target mRNA in an m^6^A-dependent manner. Recently, another class of m^6^A reader has been identified, named proline-rich coiled-coil 2A (Prrc2a), which showed preferred binding to methylated probe and stabilized a critical m^6^A-modified transcript required for myelination. Still, it is unclear how m^6^A readers selectively bind m^6^A sites or certain m^6^A-modified transcripts. Probably reader proteins are localized to the different regions of mRNA *via* interaction with other RBPs that recognize distinct features of RNA (Huang et al., 2018; Wu et al., 2019).

Heat shock stress and viral infection can cause cytoplasmic YTHDF proteins redistribution to the nucleus. It has been shown that within hours of heat shock stress, YTHDF2 gets highly upregulated at both transcript and protein levels and then the majority of the cytoplasmic YTHDF2 translocates to the nucleus (Zhou et al., 2015).

## Role of m^6^A During Virus Infections

m^6^A modification in different pathogenic viruses has been increasingly being explored in recent years where numerous reports have revealed m^6^A modification to regulate the viral life cycle. m^6^A was identified in Influenza A virus (IAV) (Krug et al., 1976), Rous Sarcoma Virus (RSV) (Kane and Beemon, 1985), B77 Avian Sarcoma Virus (Stoltzfus and Dimock, 1976; Dimock and Stoltzfus, 1977) and Feline leukemia Virus (Thomason et al., 1976) decades ago. But the specific functions and mechanisms of m^6^A in RNA viruses remained to be characterized for a long time until recent advancement in new technologies of transcriptome-wide mapping and sequencing. In the following sections, we will summarize the recent studies addressing the role of functional m^6^A modifications in viral transcripts and genomic RNA of human viruses in the context of replicative viral life cycles and associated disease pathogenesis.

## RNA Viruses

### Human Immunodeficiency Virus I

At least four major studies reported the involvement of m^6^A during human immunodeficiency virus I (HIV-1) infection (Lichinchi et al., 2016a; Tirumuru et al., 2016; Kennedy et al., 2017; Lu et al., 2018). Lichinchi et al. first reported the presence of m^6^A methylation on HIV-1 RNA and characterized the function, topology, and molecular features of m^6^A modification in viral RNA (Lichinchi et al., 2016a). At least 14 methylation peaks were identified in coding and noncoding regions, splicing junctions, and splicing regulatory sequences. It was shown that m^6^A modification levels increased in both host and viral mRNAs upon infection and this modification promoted HIV-1 replication. m^6^A modification on HIV-1 RNA positively affected the interaction between HIV-1 Rev protein and Rev response element (RRE) RNA. This phenomenon promoted the formation of Rev-RRE complex and enhanced the nuclear export of viral RNA and increased viral replication (Lichinchi et al., 2016a). Kennedy et al. identified m^6^A residues in the 3’UTR of HIV-1 RNA and found that m^6^A modification facilitated enhanced mRNA expression and virus replication (Kennedy et al., 2017). Tirumuru et al. identified m^6^A peaks in the 5’ and 3’UTR of HIV-1 RNA and noted that m^6^A modification increased HIV-1 gag protein expression and YTHDF1–3 proteins inhibited HIV-1 infection by blocking viral reverse transcription and supported viral RNA degradation (Tirumuru et al., 2016). YTHDF proteins and HIV-1 Gag protein were shown to form a complex with viral RNA (Lu et al., 2018). However, there were discrepancies between these studies due to different experimental conditions and use of different mapping techniques. The differences between these studies requires further research. Overall, m^6^A of HIV RNAs impacts profoundly the RNA functions and various aspects of HIV life cycle.

### Flaviviruses (HCV, ZIKV, DENV, WNV, and YFV)

m^6^A modification sites have been identified in approximately 19 regions of the Hepatitis C virus (HCV) RNA genome (Gokhale et al., 2016). m^6^A methyltransferases (METTL3/14) decreased the extracellular HCV RNA and production of virus particles. YTHDF proteins negatively regulated HCV virus particle production and re-localized the HCV genome to lipid droplet to inhibit viral particle assembly *via* interacting with the m^6^A-modified envelope region of the HCV genome. Mutation of m^6^A sites on the HCV E1 region dramatically increased virus production suggesting their role in virion production (Gokhale et al., 2016). During HCV infection, cellular m^6^A machinery was found to be involved with innate immune response. Modified nucleotides in HCV RNA affected the RIG-I signaling pathway (Durbin et al., 2016). HCV infection affected the m^6^A abundancy of host RNAs, including phosphatase and tensin homolog (PTEN) mRNA. HCV infection induced m^6^A modification of PTEN mRNA thus destabilized its RNA stability, leading to disrupt IFN synthesis. PTEN has been known to induce nuclear import of p-IRF3, thus affecting innate immunity to virus infection (Kim et al., 2020a). Kim et al. showed that m^6^A modified nucleotide 8766 near the HCV PAMP region reduced retinoic acid-induced gene I (RIG-I) recognition *via* the sequestration of RNA by YTHDF2 protein (Kim et al., 2020b). These results demonstrate that HCV evades immune response, among other mechanisms, by regulating host m^6^A machinery.

The RNA genomes of Zika virus (ZIKV), Dengue virus (DENV), West Nile virus (WNV), and Yellow Fever virus (YFV), were also m^6^A-mapped. Interestingly, the identified m^6^A peaks were localized to similar regions among NS3 and NS5B of viral genomes, suggesting that m^6^A modifications of the *Flaviviridae* family viruses may have a conserved role in post-transcriptional regulation (Gokhale et al., 2016). YTHDF2 protein can affect the stability of ZIKV RNA to regulate viral replication (Lichinchi et al., 2016b). The mechanism of m^6^A modification regulating ZIKV replication remained to be fully characterized.

### Influenza A Virus

Although m^6^A modification on the Influenza A Virus (IAV) HA genome was identified in late 1970s but its role in infection remained undefined (Krug et al., 1976; Narayan et al., 1987). m^6^A residues were mapped in multiple locations on both the IAV mRNA and vRNA strands. Addition of m^6^A sites in IAV transcripts enhanced the viral gene expression and replication (Courtney et al., 2017). Ectopically expressed YTHDF2 protein increased IAV replication and infectious particle production. However, YTHDF1 and YTHDF3 proteins had no impact on the viral life cycle. Thus, m^6^A plays a substantial role in regulating viral gene expression during IAV infection.

### Vesicular Stomatitis Virus

m^6^A modification of Vesicular Stomatitis Virus (VSV) RNA remains to be fully characterized. In normal situations, nuclear protein DDX46 is associated with pre-mRNA splicing and prespliceosome assembly but its role switched to a negative regulator of immune response upon viral infection. DDX46 protein can bind MAVS, Traf3, and Traf6 transcripts, which encode for signaling molecules involved in antiviral response. After VSV infection, DDX46 recruits the m^6^A demethylase AlkBH5 to remove m^6^A from these antiviral transcripts. Demethylation retains these antiviral transcripts inside the nucleus and reduces their translation which consequently disrupts antiviral immune response. Knockdown of AlkBH5 remarkably increased IFN production triggered by VSV infection, thus m^6^A modification might play a negative role in VSV life cycle (Zheng et al., 2017).

### Enterovirus 71

Enterovirus 71 (EV71) transcripts contain several m^6^A peaks mostly localized in the coding regions and the expression and localization of m^6^A methyltransferases, demethylases, and YTH proteins are affected upon virus infection (Hao et al., 2019). Perturbation of the expression of METTL3, FTO, and YTH proteins can alter EV71 replication and mutation in the m^6^A site of viral RNA significantly reduced viral replication. Host METTL3 protein interacts with the viral polymerase 3D to enhance its sumoylation and ubiquitination, which facilitates the stability of 3D and increased viral replication. Thus, m^6^A modifications in EV71 transcripts played a positive role in viral replication.

### Pneumoviruses

Human metapneumovirus **(**HMPV) causes acute respiratory infection in children, immunocompromised patients, and older individuals (Shafagati and Williams, 2018). m^6^A sites have been identified in HMPV genome, antigenome, and viral mRNAs. Five m^6^A peaks have been identified throughout the P and G regions of genomic RNA. m^6^A modification promoted HMPV replication and gene expression, whereas its abrogation resulted in the attenuation of HMPV. m^6^A-deficient HMPV RNA induced significantly higher Type I IFN in a RIG-I-dependent manner, suggesting that m^6^A modified RNAs are less sensitive to RIG-I (Lu et al., 2020). Thus, m^6^A modification served as a molecular marker for innate sensing by cells to discriminate self from non-self RNA. m^6^A modifications have been identified in Human respiratory syncytial virus (RSV) RNAs, which upregulated viral replication and pathogenesis (Xue et al., 2019).

## DNA Viruses

### Hepatitis B Virus

Hepatitis B virus (HBV) infection is one of the leading causes of chronic hepatitis that is associated with the elevated risk of severe liver disease including cirrhosis, fibrosis, and primary hepatocellular carcinoma (Ganem and Prince, 2004). HBV contains DNA genome but amplifies by reverse transcription of an intermediate pre-genomic RNA (pgRNA) (Seeger and Mason, 2015; Hu et al., 2019). Role of m^6^A has been defined in HBV transcripts and liver tissues collected from chronic HBV patients. A single m^6^A consensus DRACH motif (GGACA) was identified which resides within the epsilon stem-loop region (A1907) of all the HBV transcripts. This motif is repeated twice in the 5’ and 3’ ends of the pgRNA due to terminal redundancy but resides only once in the 3’ UTR of the subgenomic transcripts. m^6^A site located in the 3’ UTR of HBV transcripts reduced their stability, affecting corresponding viral protein expression. m^6^A reader YTHDF proteins were found to bind HBV RNAs and silencing of these m^6^A readers increased HBV protein expression by enhancing the stability of HBV RNA and similar results were observed when the m^6^A site was mutated within the 3’ epsilon loop of all HBV transcripts. m^6^A site at the 5’ epsilon stem-loop of viral pgRNA positively regulated the reverse transcription activity. Thus, m^6^A modification exerts a unique dual regulatory role during HBV life cycle, wherein it negatively regulates the stability of viral transcripts but positively affects the reverse transcription of the core-encapsidated pgRNA (Imam et al., 2018).

IFN treatment of virus-infected cells leads to a reduction of replication mostly by the degradation of viral RNAs by an IFN-stimulated gene 20 (ISG20) exonuclease (Liu Y. et al., 2017). IFN-α induced ISG20 targets the m^6^A site in the HBV RNAs (Imam et al., 2020). ISG20 formed a complex with m^6^A reader YTHDF2, in which recruited ISG20 degraded m^6^A containing HBV transcripts. Mutation in m^6^A sites abrogates ISG20 mediated RNA decay. This work identified an unknown role of m^6^A modification of HBV RNA in IFN-α-induced viral RNA degradation and proposes a new role of YTHDF2 protein as a cofactor required for IFN-α mediated HBV RNA degradation (Imam et al., 2020). 5’ epsilon stem-loop structure of HBV pgRNA is recognized by RIG-I for IFN synthesis (Sato et al., 2015). Mutation of m^6^A site at 5’ epsilon stem-loop structure of HBV pgRNA increases RIG-I sensing, while the presence of m^6^A reduces RIG-I binding affinity (Kim et al., 2020b). Thus, m^6^A modification of HBV RNA may function as a molecular signature for distinguishing self from non-self RNA *via* the RNA sensor RIG-I. HBV dramatically enhances the m^6^A modification of host PTEN RNA among others, which leads to its degradation with a corresponding decrease in PTEN protein levels (Kim et al., 2020a). PTEN, being a tumor suppressor, its low expression may contribute to hepatocarcinogenesis associate with HBV infection.

### Kaposi’s Sarcoma-Associated Herpesvirus (KSHV)

KSHV completes its life cycle in two separate phases; latent infection and lytic replication (Zhao et al., 2015; Liu L. et al., 2017). Most of the viral genome is suppressed through DNA methylation, repressive histone modifications, and other regulatory mechanisms during the latent condition of KSHV infection (Pantry and Medveczky, 2009; Gunther and Grundhoff, 2010; Lu et al., 2010; Toth et al., 2010; Purushothaman et al., 2015). Viruses can reactivate from latency to lytic cycle when the host cell microenvironment change and the inhibitory epigenetic marks are replaced by active ones to allow the transcription of viral lytic genes (Ye, 2017; Uppal et al., 2018). m^6^A modification was identified in KSHV transcripts and m^6^A modified mRNA levels significantly increased upon stimulation for lytic replication (Ye et al., 2017). METTL3 and YTHDF2 proteins promoted KSHV virion production (Hesser et al., 2018). Although opposing results showed m^6^A pathway to inhibit viral gene expression (Tan et al., 2018). By facilitating the degradation of viral transcripts, YTHDF2 protein inhibited KSHV lytic replication. Thus, m^6^A modifications play a critical role in KSHV life cycle.

### Simian Virus 40 (SV40)

SV40 is a member of the Polyomavirus family and its gene expression is regulated in an early phase (encoding the regulatory proteins) and a late phase (encoding the structural proteins). Although m^6^A modification was reported in SV40 early in 1979, but the specific location of these m^6^A sites was only recently identified (Canaani et al., 1979). Two m^6^A sites were identified on viral early transcripts and eleven m^6^A sites on the late mRNAs. Mutating the m^6^A sites on late transcripts inhibited SV40 replication, without affecting the mRNA splicing. Similarly, mutational inactivation of the YTHDF2 or METTL3 gene showed a decrease in viral replication (Tsai et al., 2018). Overall m^6^A positively regulates SV40 life cycle.

### Epstein - Barr Virus (EBV)

As a member of the Herpesvirus family, EBV was first isolated and identified from a Burkitt’s lymphoma patient in 1964 (Epstein et al., 1964). m^6^A sites have been identified on EBV latent and lytic transcripts, where the latent gene expression was enhanced and lytic gene expression was repressed (Lang et al., 2019). m^6^A methyltransferase METTL14 was dramatically increased during EBV latency while it was reduced during the lytic cycle. Also, METTL14 facilitated the cellular proliferation and colony formation of EBV transformed cells *in vitro* and increased the oncogenesis of EBV mediated tumorigenicity *in vivo*. Viral-encoded latent oncoprotein EBNA3C expression was upregulated by METTL14 mediated m^6^A modification, which is critical for the viral-mediated transformation of cells, and its expression led to a feedback loop by which METTL14 transcription was also induced, along with its protein stability. Latent antigens of EBV are the major contributors to EBV-associated malignancies and their reduced expression attenuates EBV-mediated tumorigenesis. Therefore, targeting METTL14 might be a plausible therapeutic strategy for treating EBV-associated cancer. Overall, m^6^A modification promoted growth and proliferation of EBV infected cells.

### Human Cytomegalovirus (HCMV)

HCMV usually does not initiate any complications in the healthy individual; however, in immunocompromised patients, fetuses, and neonates, HCMV infection can cause an array of damaging clinical outcomes (Crough and Khanna, 2009). m^6^A machinery is required for HCMV propagation. In the absence of METTL3 followed by viral infection resulted in the modular and highly specific hundreds of IFN-stimulated genes (ISGs) production. Drug-induced blocking of IFN signaling restored viral proliferation in METTL3- and YTHDF2-depleted cells. Even, when the cells were stimulated with an ultraviolet-inactivated virus, the same modular ISG induction was also seen, which means this effect was not driven by viral mechanisms. Central cytokine (IFN-β) that drives type I IFN response is encoded by the gene *IFNB*. The mRNA of *IFNB* was modified by m^6^A and following depletion of METTL3 and YTHDF2, it was highly stabilized. Mice lacking the m^6^A reader YTHDF3, exhibited increased *Ifna* and *Ifnb* induction upon infection with virus. Here, m^6^A modification served as a negative regulator of IFN response by controlling the fast turnover of IFN mRNAs and consequently accelerates viral proliferation (Winkler et al., 2019).

## Discussion and Future Directions

In this review, we have discussed the recent advances in identifying the role of m^6^A methylation in viral life cycles, including both the RNA and DNA viruses. m^6^A modifications were either supportive (proviral) or obstructive (antiviral) during viral infections, suggesting a complex pattern of epitranscriptomic regulation of viral gene expression. m^6^A modification affected viral life cycle in a wide variety of ways ultimately affecting disease pathogenesis. There were mechanistic inconsistencies in some of these studies reflecting different conclusions. These disparities may have originated from the use of different cell lines and experimental procedures of analyses. RNA methylation adds a new layer of regulation of RNA functions. m^6^A modification is a dynamic process that occurs co-transcriptionally as nascent mRNAs are being transcribed. Studies have shown that METTL3/14 methyltransferases are associated with open chromatin, the sites of transcription initiation (Barbieri et al., 2017; Knuckles et al., 2017; Xiang et al., 2017; Bertero et al., 2018; Huang et al., 2019). METTL enzymes have been localized both in the cytoplasm and nucleus. In cancer cells, the cytoplasmic METTLs have been found while in primary cells, it is mostly localized in the nucleus. For RNA genomes of RNA viruses such as HCV, ZIKA virus and others, RNA methylation likely occurs in the cytoplasm associated with replication complexes formed on organellar membranes. What other functions are associated with cytoplasmic methyltransferases in eukaryotic cells remains to be characterized. Various cellular signals may trigger the function of m^6^A in different ways in different cell lines. Subcellular localization of m^6^A writer, eraser and reader proteins and their associated binding partners may affect the biological events in different ways. Distinct reader proteins target different transcripts in different cells or tissues. Even the location of m^6^A site in certain transcript may have variable consequences in regulatory functions.

N6 methylation of viral RNAs provides potential antiviral therapeutic opportunities. In this context, 3-deazaadenosine (DAA) (a methylase inhibitor) and meclofenamic acid (MA) (an FTO inhibitor) have been tested. DAA can decreases the formation of S-adenosylmethionine (SAM) without affecting mRNA capping and reduces mRNA cap methylation and all types of SAM-dependent RNA methylations (Fustin et al., 2013). A wide variety of viruses have been suppressed by DAA (Bader et al., 1978; Fischer et al., 1990; Wyde et al., 1990; Mayers et al., 1995; Bray et al., 2000; Gordon et al., 2003; Courtney et al., 2017; Kennedy et al., 2017). Since, DAA inhibits all types of RNA modifications, it is difficult to conclude whether antiviral effect comes from inhibition of mRNA cap methylation or internal m^6^A methylation. As such currently, there is no drug that can specifically inhibit m^6^A. FTO inhibitor, MA has been reported to enhance the expression of KSHV lytic genes, by competing with m^6^A-containing substrate binding and inhibition of FTO driven demethylation (Ye et al., 2017). Therefore, the development of novel small molecule inhibitors or drugs specifically targeted for m^6^A machinery (writers, erasers, or readers) would be a highly significant venue for antiviral therapies.

Since m^6^A deficient HMPV can induce higher IFN production, live attenuated vaccine candidates for HMPV and other pneumoviruses were designed (Lu et al., 2020). Similar approaches can be applied to other viruses for the rational design of vaccine candidates. Apart from m^6^A modification, mammalian epitranscriptome contains 3 other internal modifications, Pseudouridine (ψ), N^1^-methyladenosine (m^1^A), and 5-methylcytosine (m^5^C) (Roundtree et al., 2017a). Future research can be expanded on these less explored RNA modifications to gain adequate information on their role in viral RNAs regulation and cellular antiviral responses.

## Author Contributions

AS and HI conceptualized and wrote the manuscript. G-WK contributed to writing. All authors contributed to the article and approved the submitted version.

## Funding

National Institutes of Health NIH AI139234.

## Conflict of Interest

The authors declare that the research was conducted in the absence of any commercial or financial relationships that could be construed as a potential conflict of interest.
